# Giant scrotal lipoma in Madelung's disease: A case report

**DOI:** 10.1016/j.ijscr.2023.108151

**Published:** 2023-04-13

**Authors:** Pham Thi Viet Dung, Tran Thiet Son, Truong Quoc Son, Nguyen Minh Tuan

**Affiliations:** a Department of Plastic and Reconstructive Surgery, Bach Mai Hospital, Hanoi, Viet Nam; b Faculty of Plastic and Reconstructive Surgery, Hanoi Medical University, Hanoi, Viet Nam; c Department of Plastic and Reconstructive Surgery, Hanoi Medical University Hospital, Hanoi, Viet Nam; d Urologic Surgery Department, Bach Mai Hospital, Hanoi, Viet Nam

**Keywords:** Giant tumor, Scrotal tumor, Madelung's disease, Case report

## Abstract

**Introduction and importance:**

Madelung disease is a rare condition of unknown etiology, characterized by large masses of subcutaneous fat in the upper body bilaterally. It rarely affects the lower extremities and genital region.

**Case presentation:**

Here, we report a patient with Donhouser's type III Madelung's disease. A 47-year-old male patient presented with a giant fatty scrotal tumor that caused deformation of the scrotum and penis, made it difficult to perform daily activities, and hindered sexual activity. The adipose tumor was completely removed using a midline scrotal incision. The scrotum was reconstructed with bilateral anterior and posterior scrotal skin flaps. The excess skin was cut into a wedge shape between the anterior and posterior scrotal regions.

**Clinical discussion:**

At 3 months postoperatively, the scrotum was normal in shape and size, and the patient was able to perform personal activities and normal sexual activity. The surgical options, lipectomy results, and experiences drawn from the clinical cases have been discussed.

**Conclusion:**

Giant scrotal lipomas are very rare in Madelung's disease. Lipectomy and scrotal reconstruction are required. Wedge-shaped scrotal skin excision in the midsection on each side of the scrotum removes excess skin, which could restore the shape and function of the penis and scrotum.

## Highlights

• A giant lipoma with a net weight of 1650 g
• Giant scrotal lipoma is very rare in Madelung's disease.
• Reconstructing scrotum after tumor resection

## 1 Introduction

Multiple symmetric lipomatosis (MSL) is called Madelung disease, Madelung was first described by Benjamin Brodie in 1846, and subsequently reported by Otto Madelung in 1888 and Launois Bensaude in 1898 [1]. This is a rare disease with an incidence of approximately 1 in 25,000 people [2]. The disease is characterized by the presence of symmetrical non-enveloped adipose masses, mainly in the upper body (face, neck, upper trunk) [2], [3], which are benign and rarely malignant [4], [5]. The disease primarily causes cosmetic disfigurement. Madelung disease rarely causes complications; the enlarged fat deposits cause compression symptoms, such as dyspnea and dysphagia due to tracheal and esophageal compression and hoarseness of voice secondary to nerve compression [6], [7]. MSL seldom affects the lower extremities and genital region. Here, we report a patient with Madelung type III who presented with multiple, symmetrical, adipose tissue masses in the upper half of the body and the scrotum. A large fatty tumor was located in the scrotum, causing deformation of the scrotum and penis. The surgical methods, outcomes, and experiences are discussed here. The work has been reported in line with the SCARE criteria [8].

## 2 Case presentation

**Fig. 1:**
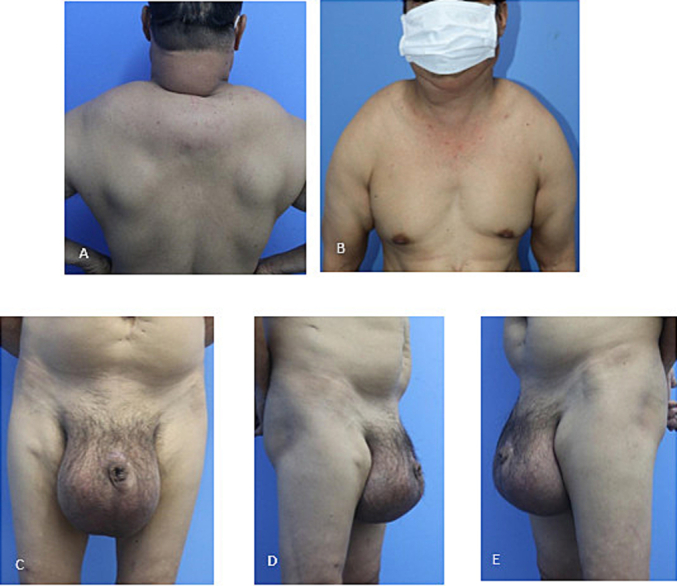
Preoperative images. (A) Multiple fatty lesions in the nape, shoulders, and upper back. (B) Multiple lesions in the anterior neck, supraclavicular fossa, and bilateral arms. (C)(D)(E) Giant tumor causing deformity of the scrotum and penis.

**Fig. 2:**
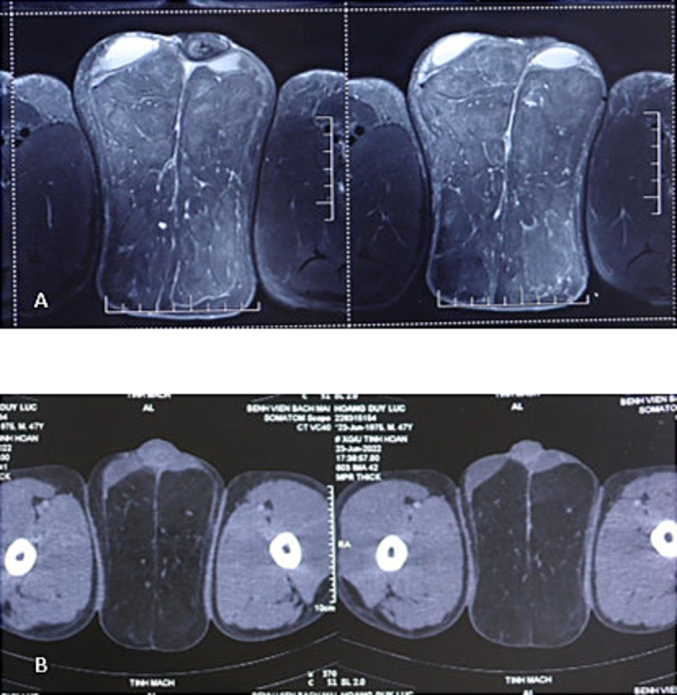
MRI images of the scrotal lesion. (A) T1- and T2-weighted coronal images showing high-signal intensity for fat deposition. The testes are pushed forwards and septae are seen. (B) T1 FS-weighted coronal images showing low-signal intensity for fat deposition.

A 47-year-old male, who hospitalized due to a giant scrotal tumor. He has a history of chronic alcoholism, developed numerous soft masses in the anterior neck, supraclavicular fossa, nape, bilateral arms, and shoulders 10 years ago. The tumors were painless and gradually enlarged in size. Five years ago, the patient developed a soft mass in the scrotum. The scrotal mass rapidly increased in size, he complained that hindering his ability to have sexual activity. Furthermore, the patient found it difficult to perform his daily activities. A physical examination revealed masses in the anterior neck, nape, shoulder, upper back, and scrotum. The mass was soft and poorly defined. The largest one was the scrotal swelling, causing expansion of the scrotal skin and buried penis (Fig. 1). The penis could not be palpated if it was not erect; a short portion of the penis protruding to the surface could be manipulated during an erection, the prepuce and meatus were not abnormal. Normal-sized testicles were palpable in addition to the tumor, which moved independent of the tumor. His BMI was 26. Magnetic resonance imaging (MRI) revealed non-encapsulated adipose tissue on either side of the scrotum (Fig. 2). An excision biopsy revealed a benign lipomatous tissue. Thyroid ultrasound image and thyroid hormones was normal. The levels of lipid in the blood, including cholesterol and triglycerides were within normal range. Liver function test results were abnormal. The levels of factors II, V, and VII were decreased, which in turn reduced the thrombocyte count and PTT levels. Thus, the patient was treated preoperatively with infusions of fresh frozen plasma and vitamin K.

**Fig. 3:**
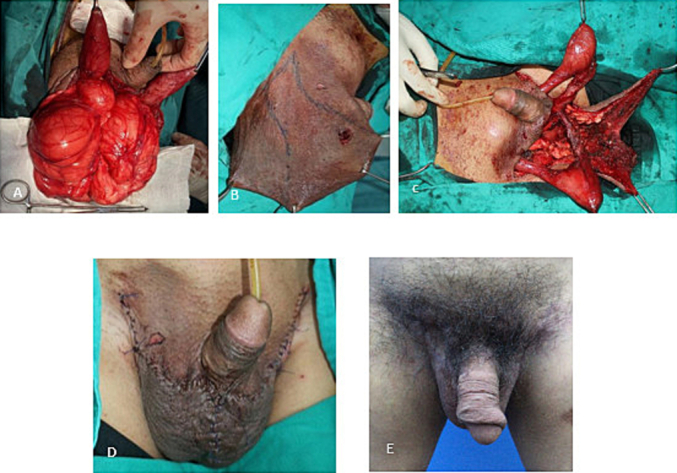
Perioperative images during excision of the lipomatous mass in the scrotum. (A) The tumor was exposed. (B) A wedge-shaped projection of the excess skin to be removed was drawn. (C) Scrotal flap with lower stalk. (D) Penis and scrotum immediately after surgery. (E) Three months after surgery.

An incision was made at the midline of the scrotal skin, and the tumor was dissected from the scrotal skin and surrounding tissues. The bilateral epididymis, spermatic cord, and testes were preserved. The lower part of the tumor could be easily dissected from the scrotal subcutaneous tissue. However, it was firmly attached to the scrotal skin. The resected fatty tumor was pale yellow, multi-lobed, and weighed 1650 g. The excess skin was excised in a wedge shape between the anterior and posterior scrotal regions to preserve the flap supplied by the anterior and posterior scrotal arteries (Fig. 3). The patient was advised for quitting alcohol, weight loss after operation. At 3 months postoperatively, the patient confessed that he was able to perform normal sexual activity and his quality of life was improved.

## 3 Discussion

Madelung's disease is a rare condition with an incidence of approximately 1 in 25,000 patients. The disease is more commonly encountered in Mediterranean countries than the rest of the world and predominantly affects men between 30 and 60 years of age (male:female ratio = 15:1) with a history of alcoholism [1], [9]. Special cases have also been described in children [1]. Several symmetrical lipomas primarily concentrated in the upper body, and sometimes in the genital area, buttocks, or extremities, can be detected on clinical and ultrasonographic examinations. CT or MRI results reinforce the diagnosis, help determine the boundaries, and identify compression of the adjacent structures. If the tumor is too large or if there are unusual manifestations (fluid, ulcer, etc.), histopathological examination should be performed to determine the nature of the tumor. The differential diagnoses of MSL include obesity, Cushing's syndrome, angiolipomatosis, encapsulated fibromas, neurofibromatosis, myxoid liposarcoma, lymphoma, salivary gland disease, Frölich syndrome and lipomatosis in patients with HIV. If the lipoma is located in the scrotum, it should be differentiated from an inguinal hernia, testicular or spermatic cord tumor, varicocele, or spermatic cord cyst. In 1984, Enzi classified MSL according to the site of fat overgrowth [10]. MSL type I is characterized by lesions distributed predominantly in the parotid gland, neck, submental region, shoulders, supraclavicular triangle and proximal upper limbs. MSL type II is characterized by diffuse lipomatous tissue deposition in the subcutaneous layer of the abdomen and thighs. Its appearance resembles the fat distribution seen in obesity. In 1991, Donhauser et al. added a type III or gynecoid type to Enzi's classification, which involves fat deposition predominantly in the pelvic region, as seen in this patient [11], [12]. A search of authenticated databases, such as PubMed and Web of Science, for ‘Multiple symmetric lipomatosis’ and ‘Madelung disease’ reveled only eight cases that were surgically treated. Of these eight patients, one complained of abnormal urination, two suffered from sexual dysfunction, two had hidden penis syndrome, and one had unilateral testicular atrophy [1], [13], [14].

Madelung's disease is clearly associated with alcohol abuse. Although alcohol cessation and weight loss are recommended, they are not effective in reversing or stopping disease progression. In addition, medical treatment is ineffective [2], [11]. Spontaneous regression of lipomas does not occur [10]; and lipectomy and liposuction are the only effective treatment options [1], [11]. Liposuction is indicated for small lesions. The main advantage of liposuction is that it reduces the risk of morbidity in obese patients, heavy smokers with high alcohol intake, and patients with severe liver disease and other metabolic disorders. Compared with the open approach, this technique results in a smaller scar, shorter recovery period, and lower surgical cost and complication rates [1]. However, liposuction cannot be performed for giant lipomas and in areas closely related to important anatomical structures such as blood vessels, nerves, testes, and spermatic cord. Furthermore, it can be difficult to perform liposuction in patients whose lipoma has a fibrous stroma. Lipectomy is more effective in patients with severe cosmetic deformities and should be considered the first choice of treatment when nerves and major vessels are involved.

According to the literature, the largest lipoma weighed 990 g [1], [13], [14]. The lipoma excised in this case is 1650 g. Hence, this can be considered a giant lipoma. In the case of very large scrotal lipomas, removal of the tumor and excess skin without damaging adjacent tissues is difficult. A midline scrotal incision is necessary for the easy dissection of a giant lipoma on both sides of the scrotum. After total removal of the fatty tumor, the excess skin should be resected in a wedge shape between the anterior and posterior scrotal regions. This is done because: 1) it removes the excess skin both horizontally and vertically, and 2) the scrotum is well-supplied by the anterior scrotal artery from the external iliac artery and the posterior scrotal artery from the internal iliac artery, which are often terminal branches of the scrotal subcutaneous plexuses [15], [16]. Therefore, the skin between the two areas should be excised as that area could have the poorest blood supply.

## 4 Conclusion

A giant scrotal lipoma is rare in Madelung's disease. Lipectomy and scrotal reconstruction are required. Excision of wedge-shaped scrotal skin in the midsection on both sides of the scrotum removes excess skin, which could restore the shape and function of the penis and scrotum.

## Consent

Written informed consent was obtained from the patient for publication of this case report and accompanying images. A copy of the written consent is available for review by the Editor-in-Chief of this journal on request.

## Ethical approval

We declare that our institution/hospital/university does not require ethical approval of clinical case reports.

## Funding

The authors have no associations or financial disclosures to report that create a conflict of interest with the information presented in this article.

## Author contribution

Pham Thi Viet Dung: senior author, operating, consulting, looking after patient, conceptualization, writing original draft, review & editing.

Truong Quoc Son: writing the original draft.

Nguyen Minh Tuan: operating surgeon.

Tran Thiet Son: review & editing.

Approval of final manuscript: all authors.

## Guarantor

Prof. Tran Thiet Son.

## Research registration number

N/A.

## Declaration of competing interest

The authors have no associations or financial disclosures to report that create a conflict of interest with the information presented in this article.
